# How to prescribe spectacles for near vision

**Published:** 2019-12-17

**Authors:** Sue Stevens

**Affiliations:** 1 Former Nursing Advisor: *Community Eye Health Journal*, International Centre for Eye Health, London School of Hygiene & Tropical Medicine, UK.

**Many people aged 40 years and above need near vision spectacles for reading and other essential daily tasks.**

As we grow older, the lens loses the ability to focus at close distances. Starting around the age of 40, near vision will slowly become worse, but distance vision will not be affected; this is known as presbyopia.

## Indications

People with presbyopia usually say that their near vision has slowly become worse.

## You will need

- Distance and near vision charts with letters, Es or shapes
- Pinhole (optional)
- A trial set of lenses or a selection of ready-made spectacles (RMS). Most people with presbyopia do not need spectacles with powers of less than +1.00 or more than +3.00. See Table 1 for suggested powers.

**Table 1:** Suggested lens powers for correction of presbyopia

+1.00	Weaker power
+1.50	
+2.00	
+2.50	
+3.00	
+3.50	Stronger power

## History

Before prescribing spectacles for presbyopia, take a careful history (pp. 44–45) and carry out a comprehensive eye examination to make sure there is nothing else wrong with the person's eyes.

## Examination

### 1 Measure the distance vision in each eye

- If the presenting vision is 6/12 or worse in either eye, find out the cause of poor distance vision before prescribing spectacles for near vision.
- If the distance vision is 6/9 or better in each eye then one can proceed with checking near vision.

### 2 Assess working distance

**Figure 1:**
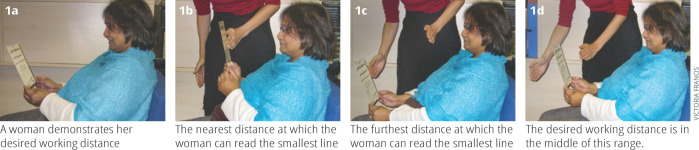
Finding the right prescription for presbyopia

- The correct power of spectacles for presbyopia depends on the person's age, the distance at which they want to see for near work, and how well they can see.
- Find out the person's working distance; this is the distance at which they would like to do most of their near work (see Figure 1a).
- Ask him or her to hold a near vision chart at the distance they do most near tasks. Around 40 cm is a comfortable distance for most people.

### 3 Measure near vision

- Ask the person to hold the chart at the distance they want to see clearly (the desired working distance) with both eyes open. Ask them to read the smallest line or show the smallest shapes they can see clearly. Write this down as their near visual acuity (e.g., N6 or J6).
- If the person already has spectacles for presbyopia, measure their near vision with these being worn. Write this down as ‘near visual acuity with spectacles'
- If the person is able to see N6 or better without any spectacles, they might not need spectacles for presbyopia. If they can see N6 or better with their old spectacles, they might not need new spectacles.

### 4 Identify the correct lens power

- Use the person's age as an idea for what power of near lens they may need. Table 2.
  **Table 2:** Suggested lens power for different ages

Person's age	Lens power
35 to 45	+1.00
46 to 50	+1.50
51 to 55	+2.00
Over 55	+2.50 or higher
- While the person wears spectacles with the selected power (or trial lenses of the same number), give them the near chart again and ask them to hold it at the desired working distance (Figure 1a). If the person cannot see at least the N6 line, try again with the next stronger power until they can see the N6 line.
- Ask the person to look at the smallest line they can see on the near chart with the near lenses, and then bring the chart closer until the letters become blurred. Hold one hand to mark the nearest distance (Figure 1b), then ask the person to move the chart further away until the letters become blurred. Mark the furthest distance (Figure 1c). This is the range of clear vision available to the person while wearing the selected lens power.
- Ask the person again to hold the chart at their desired working distance. If the range is correct, the working distance should be in the middle of this range, for example at about 40 cm (Figure 1d). This means that a person will be able to see clearly for the same distance in front and behind their working distance.

### 5 Prescribe and dispense spectacles

- Prescribe and give the reading spectacles.
- Ensure that the patient understands they are only for reading and not for seeing in the distance.
- Advise the patient that a good reading light will help to improve their near vision.

